# Risk factors for denosumab-related osteonecrosis of the jaw: a pharmacovigilance study using the U.S. FDA adverse event reporting system database

**DOI:** 10.3389/fendo.2025.1586626

**Published:** 2025-12-19

**Authors:** Hehe Bai, Jinping Wang, Xiaonian Han, Huan Li, Xiaojing Nie, Guan Wang

**Affiliations:** 1 Department of Pharmacy, Xi’ an Central Hospital, Xi’an, Shaanxi, China; 2 School of Pharmaceutical Sciences, Tsinghua University, Beijing, China

**Keywords:** denosumab, osteonecrosis of the jaw, risk factors, concomitant medications, time-to-onset, FAERS database

## Abstract

**Objective:**

This study aims to comprehensively evaluate risk factors for denosumab-related osteonecrosis of the jaw (ONJ), focusing on dosage, concomitant medications, and patient demographics, to inform clinical decision-making and optimize therapeutic strategies.

**Methods:**

A retrospective pharmacovigilance analysis was conducted using data from the US FDA Adverse Event Reporting System (FAERS) spanning Q2–2010 to Q3 2024. Disproportionality analysis, logistic regression analysis, and time-to-onset analysis were employed to assess the association between denosumab use and ONJ. Key variables included dosage (60 mg Q6m *vs*. 120 mg Q4w), concomitant medications (e.g., bisphosphonates, glucocorticoids, chemotherapies), and patient demographics (age, sex).

**Results:**

Among 7,689 denosumab-related ONJ cases, the 120 mg dose demonstrated markedly higher ONJ risk (reporting odds ratio [ROR] = 245.47 *vs*. 28.59 for 60 mg; *P* < 0.001), with a 54-day shorter median onset time (452 *vs*. 506 days; P = 0.011). Combination therapies synergistically amplified risk, with sequential administration of zoledronic acid yielding the highest ROR at 726.43 (95% CI: 642.83–820.90) and an accelerated time to onset (median 378 days *vs*. 462 days for monotherapy). Multivariate logistic regression confirmed age ≥65 years (adjusted odds ratio [aOR] = 1.48), high-dose denosumab (aOR = 7.18), and concomitant medications (e.g., bisphosphonates, glucocorticoids, chemotherapies) as independent risk factors. Male patients had a threefold higher risk than females (ROR = 231.11 *vs*. 51.01), while age showed minimal influence on latency periods (*P* = 0.808). Weibull analysis demonstrated wear-out failure (β>1) in most subgroups, indicating cumulative toxicity; however, random failure patterns (β≈1) were observed with: 60 mg dosing and either sequential therapies (alendronate/ibandronate/risedronate) or combination therapy with methotrexate.

**Conclusions:**

This study demonstrates that the risk of denosumab-related ONJ is multifactorial, driven by high-dose regimens (120 mg Q4w), specific polypharmacy (particularly concomitant bisphosphonates and glucocorticoids) and male sex. These findings underscore the need for prospective validation and mechanistic investigation into the underlying drug interactions.

## 1 Introduction

Denosumab, a fully human monoclonal antibody, exerts its therapeutic effect by selectively binding to the receptor activator of nuclear factor kappa-B ligand (RANKL), thereby inhibiting osteoclast formation, function, and survival. This mechanism underpins its clinical utility in reducing bone resorption, making it a cornerstone in the management of osteoporosis, bone metastases, and other metabolic bone disorders (1). Its unique mode of action has established denosumab as a highly effective therapeutic agent for osteoporosis, bone metastases, and other metabolic bone disorders (2). Unlike traditional bisphosphonates, denosumab does not require dose adjustment based on renal function and is not associated with acute-phase reactions (3), making it a preferred alternative or adjunctive treatment in numerous international guidelines (4–7). The National Comprehensive Cancer Network (NCCN) and the European Society for Medical Oncology (ESMO) further highlight its pivotal role in preventing skeletal-related events in patients with metastatic bone disease (8, 9).

However, the clinical application of denosumab faces significant challenges due to its association with osteonecrosis of the jaw (ONJ), a severe adverse reaction. Characterized by intense pain, facial swelling, and oral dysfunction, ONJ profoundly impairs patients’ quality of life and poses a substantial public health burden (10). Multiple studies indicate that denosumab carries a higher incidence of ONJ compared to zoledronic acid (11–13), with data from the US Food and Drug Administration (FDA) Adverse Event Reporting System (FAERS) revealing that denosumab accounts for 54.5% of all drug-related ONJ cases (14), underscoring the urgency of risk mitigation strategies.

Current research on denosumab-related ONJ predominantly focuses on patient-specific factors, such as pre-existing dental conditions, diabetes, and hypertension, as well as medical interventions like tooth extraction or periodontal surgery (15–17). Regarding pharmacological determinants, dosage, injection frequency, and prior bisphosphonate exposure have been identified as risk factors (18, 19). However, the influence of co-medications remains understudied. While limited evidence suggests that therapies such as corticosteroids, chemotherapy, or targeted agents may synergistically elevate ONJ risk (17, 20), the specific mechanisms and magnitude of risk associated with drug combinations remain poorly defined, warranting further investigation.

This study leverages the FAERS database to comprehensively evaluate risk factors for denosumab-related ONJ through reporting odds ratios (RORs), time-to-onset analyses, and multivariate logistic regression. Particular emphasis is placed on the potential impact of co-medications, including anti-osteoporotic agents, anti-tumor drugs, and glucocorticoids. The findings aim to provide evidence-based insights for clinical decision-making, optimizing therapeutic strategies to balance efficacy and safety, ultimately reducing the incidence of ONJ.

## 2 Materials and methods

### 2.1 Data source

This study was designed as an observational, retrospective pharmacovigilance analysis using disproportionality methods to evaluate the potential association between denosumab-related ONJ and combination therapies. Data were extracted from the FAERS, encompassing reports from Q2–2010 to Q3 2024, with a total of 19,473,776 reports included for analysis. The FAERS database comprises anonymized, spontaneous adverse event (AE) reports submitted by healthcare professionals, patients, and pharmaceutical manufacturers, offering a comprehensive and valuable resource for pharmacovigilance research. As the database contains de-identified and publicly available data, this study was exempt from the requirement for ethical approval and patient consent. Data were extracted from the publicly available FDA Adverse Event Reporting System (FAERS) database, accessible online at https://www.fda.gov/drugs/drug-approvals-and-databases/fda-adverse-event-reporting-system-faers-database. As the database contains fully de-identified and publicly available data, this study was exempt from the requirement for ethical approval and patient consent.

### 2.2 Data cleaning

**Figure 1:**
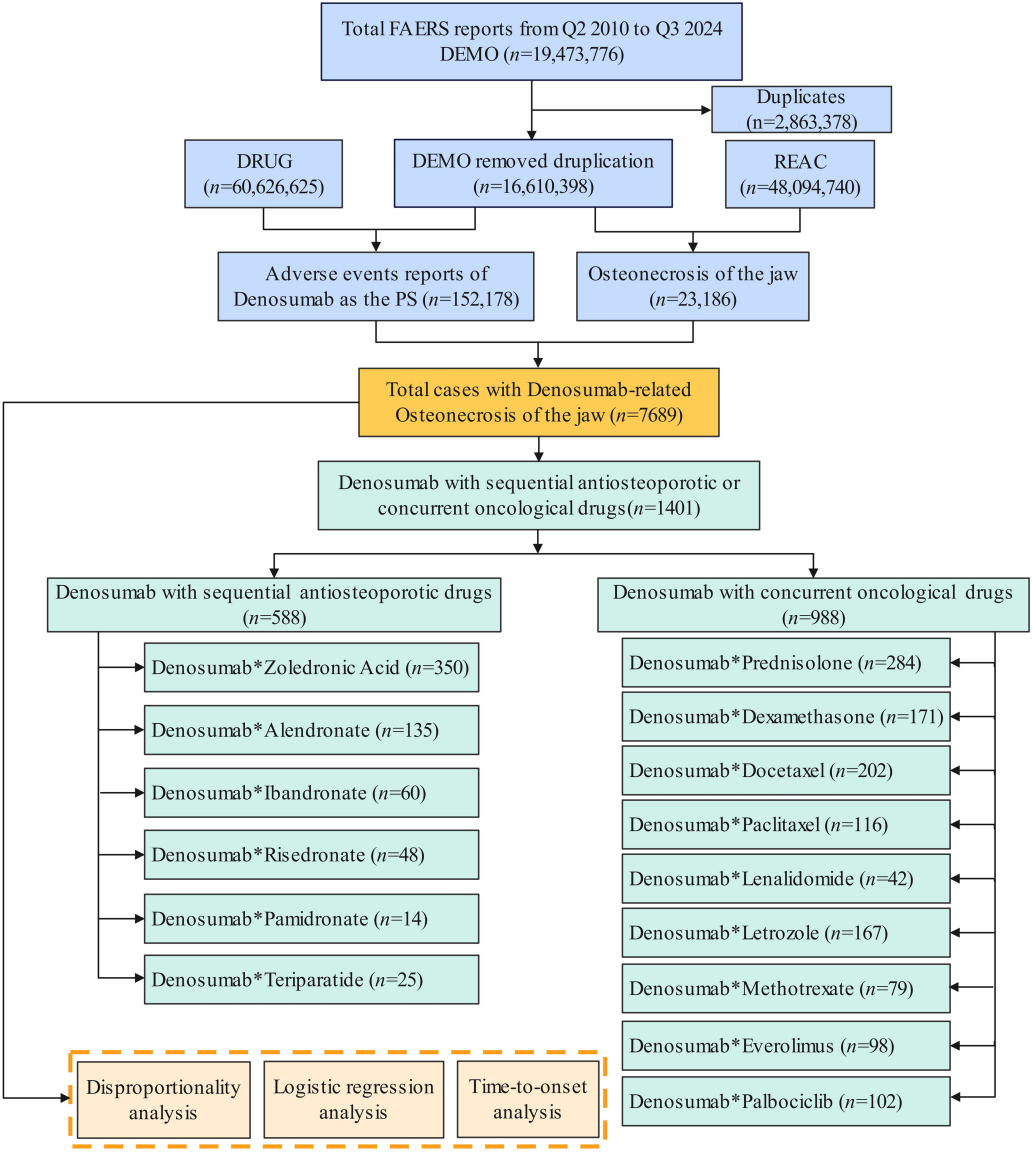
Process of screening cases of denosumab-related osteonecrosis of the jaw from FAERS database.

The data cleaning process involved several steps to ensure dataset quality and reliability. Duplicate records were removed by selecting the report with the most recent FDA receipt date (FDA_DT) when the unique case identifier (CASEID) was identical, and the higher unique primary identifier (PRIMARYID) when both CASEID and FDA_DT matched (21). Reports containing incomplete or ambiguous information—such as missing patient demographics or drug details—were also excluded. Following this procedure, the final analytical cohort comprised 16,610,398 valid reports. A detailed flowchart of the case selection process is provided in **Figure 1**.

Drug names were standardized to generic terms using the FDA’s Structured Product Labels to ensure consistency. Cases were defined as reports where denosumab was listed as the primary suspect (PS) drug in association with the preferred term (PT) “osteonecrosis of jaw”. All other medications listed in these case reports were analyzed as concomitant medications. Notably, as the FAERS database does not reliably capture temporal drug administration sequences, the term “concomitant” here refers to drugs co-reported within the same case and may encompass both truly simultaneous use and sequential administration (e.g., prior bisphosphonate therapy followed by denosumab). AEs were mapped to preferred terms in the Medical Dictionary for Regulatory Activities (MedDRA, version 27.1), with “osteonecrosis of the jaw” (PT code: 10064658) specified as the target event for analysis (https://www.meddra.org/training/schedule/1683/What’s-New-with-MedDRA-V27.1-and-the-MSSO).

Integrated medications were classified into the following two groups to evaluate their potential association with ONJ:

- Sequential antiosteoporotic drugs: bisphosphonates (zoledronic acid, alendronate, ibandronate, risedronate and pamidronate) and the anabolic agent teriparatide.
- Concurrent oncological drugs: chemotherapeutic agents (docetaxel, paclitaxel and methotrexate), targeted biological agents (lenalidomide, everolimus and palbociclib), glucocorticoids (prednisolone and dexamethasone) and endocrine therapy (letrozole).

Analyses involving sequential antiosteoporotic drugs (bisphosphonates, teriparatide) predominantly feature the 60 mg Q6m denosumab regimen, reflecting real-world prescribing patterns for osteoporosis management. Evaluations of concurrent oncological therapies (chemotherapeutic agents, targeted therapies, glucocorticoids) primarily represent the 120 mg Q4w dosage, consistent with its licensed indication for malignancy-related bone disease.

### 2.3 Statistical analysis

#### 2.3.1 Disproportionality analysis

Disproportionality analysis is a widely used data mining approach in pharmacovigilance for identifying potential safety signals from spontaneous reporting databases (22, 23). This method is endorsed by regulatory agencies for post-marketing surveillance and has been extensively applied in studies of medication-related ONJ and other adverse drug reactions (14, 24). In this study, the ROR method was employed to evaluate potential associations between denosumab, its concomitant medications, and ONJ. “Cases” were defined as patients with ONJ linked to the target drug (denosumab or its concomitant medications), while “non-cases” comprised patients experiencing other AEs associated with the target drug. The ROR was calculated as the ratio of the odds of ONJ reports for the target drug relative to all other drugs in the FAERS database (12), using the following formula: $\text{ROR}=\frac{\text{a}/\text{c}}{\text{b}/\text{d}}$,

Where:

 a= number of reports of the target drug with ONJ,
 b= number of reports of all other drugs with ONJ,
 c= number of reports of the target drug with all other AEs,
 d= number of reports of all other drugs with all other AEs.

The 95% confidence interval (CI) of the ROR was computed as: 95% CI = ${\text{e}}^{\text{ln}\left(\text{ROR}\right)\pm 1.96\sqrt{\frac{1}{\text{a}}\;+\;\frac{1}{\text{b}}\;+\;\frac{1}{\text{c}}\;+\;\frac{1}{\text{d}}}}$. A safety signal was considered significant if the lower limit of the 95% CI > 1 and there were at least three reported cases (12).

To assess the influence of age (<65 years and ≥65 years), sex (male *vs*. female), dosage (60 mg Q6m *vs*. 120 mg Q4w), and concomitant medications (anti-osteoporotic drugs and anti-tumor drugs) on the risk of ONJ associated with denosumab, the reports were stratified and analyzed using disproportionality analysis. The ROR and its 95% CI were calculated for each subgroup to evaluate the robustness of the results.

#### 2.3.2 Logistic regression analysis

To further investigate the risk factors influencing the occurrence of denosumab-related ONJ, logistic regression analysis was conducted using all denosumab-related reports. However, prior to the analysis, a crucial step of data cleaning was implemented to handle missing values. For each variable including age (<65 years and ≥65 years), sex (male *vs*. female), dosage (60 mg Q6m *vs*. 120 mg Q4w), and concomitant medications (anti-osteoporotic drugs and anti-tumor drugs), if any of these variables had a missing value within a particular report, that entire report was removed from the dataset. This ensured that only complete and reliable data were used for the subsequent analyses. After this data cleaning process to address missing values for key variables (age, sex, dosage), the analysis proceeded by adjusting for potential confounders to enhance the validity and robustness of the findings. Univariate logistic regression analysis was initially performed to identify potential risk factors, with variables exhibiting a *P*-value< 0.20 considered statistically significant and subsequently included in the multivariate analysis (25). For both the univariate and multivariate analyses, Odds ratios (ORs) and their corresponding 95% CIs were calculated for the risk factors. This yielded crude ORs from the univariate analysis and adjusted ORs from the multivariate analysis. The significance of each variable was then assessed using the likelihood ratio test, with a two-sided P-value< 0.05 considered statistically significant (26).

#### 2.3.3 Time‐to‐onset analysis

The time-to-onset of ONJ was defined as the interval from the initiation of denosumab treatment, including its concomitant medications, to the onset of ONJ. The variation in the incidence rate of ONJ was assessed using the Weibull shape parameter (WSP) test (27). The Weibull distribution is characterized by two parameters: the scale parameter (α) and the shape parameter (β). The scale parameter (α) determines the spread of the distribution, where a larger value stretches the distribution, while the shape parameter (β) influences the shape of the distribution curve, with a larger value producing a left-skewed curve. The shape parameter (β) reflects the level of hazard over time in the absence of a reference population, as follows: if β< 1 and its 95% CI< 1, the hazard level is estimated to decrease over time (early-failure type); if was equal to or nearly 1and its 95% CI includes 1, the hazard level is considered constant over time (random-failure type); if β > 1 and its 95% CI excludes 1, the hazard level is evaluated as increasing over time (wear-out-failure type) (27, 28). The median time-to-onset, accompanied by the 25th and 75th percentiles, was presented as the median (interquartile range [IQR]). Additionally, the Weibull shape (β) and scale (α) parameters were estimated to characterize the hazard profile of ONJ. Additionally, the Mann-Whitney U test was employed to compare differences in time-to-onset between different dosages of denosumab (60 mg *vs*. 120 mg) and between patients receiving denosumab with concomitant medications versus those without concomitant medications.

In this study, results with a two-sided *P*-value of< 0.05 were considered statistically significant. All statistical analyses were performed using RStudio software (version 4.4.2).

## 3 Results

### 3.1 Case characteristics and concomitant medication use

During the study period (Q2–2010 to Q3 2024), a total of 48,094,740 AEs were recorded in the FAERS database, among which 23,186 reports were associated with ONJ. Of these, 7,689 cases were identified as denosumab-related ONJ, representing 33.16% of all ONJ cases. These denosumab-related ONJ cases were extracted from 152,178 reports where denosumab was listed as the PS drug (**Figure 1**).

Of the 7,689 denosumab-related ONJ cases, 1,401 (18.22%) involved concomitant use of anti-osteoporotic or anti-tumor drugs. Overlapping drug combinations resulted in cumulative counts exceeding total cases. Anti-osteoporotic drugs (588 cases) included zoledronic acid (n=350), alendronate (n=135), ibandronate (n=60), risedronate (n=48), pamidronate (n=14), and teriparatide (n=25). Anti-tumor drugs (988 cases) comprised docetaxel (n=202), paclitaxel (n=116), methotrexate (n=171), lenalidomide (n=167), everolimus (n=98), palbociclib (n=102), prednisolone (n=284), and dexamethasone (n=171).

The baseline characteristics of the study cohort are summarized in **Table 1**. Among the 152,178 denosumab-treated patients identified from the FAERS database, 7,689 (5.1%) experienced ONJ, while the remaining 144,489 (94.9%) reported other AEs. The majority of ONJ reports were submitted by healthcare professionals (85.0%), a significantly higher proportion than for non-ONJ reports (66.3%). A notable difference was observed in sex distribution: among reports with available sex information, males accounted for 67.2% of ONJ cases compared to 10.3% of non-ONJ reports. The median age was similar between groups. Geographically, the United States contributed the most reports for both ONJ and non-ONJ cases; however, Japan and Germany were more prominent among ONJ reports. Regarding outcomes, death and hospitalization were less frequent in ONJ cases than in non-ONJ cases, whereas disability was more commonly reported in ONJ cases.

**Table 1:** Baseline characteristics of denosumab-treated patients with or without ONJ events reported in the FAERS database.

Characteristics	All cases (N=152178)	ONJ (N=7689)	Non-ONJ (N=144489)
Reporter			
Data available	150744	7668	143076
Healthcare professional	101403(67.3%)	6520(85.0%)	94883(66.3%)
Non-healthcare professional	49341(32.7%)	1148(15.0%)	48193(33.7%)
Sex			
Data available	133866	6562	127304
Male	17535(13.1%)	4412(67.2%)	13123(10.3%)
Female	116331(86.9%)	2150(32.8%)	114181(89.7%)
Age (year)			
Data available	89486	4813	84673
0–17	249(0.3%)	5(0.1%)	244(0.3%)
18–44	1532(1.7%)	72(1.5%)	1460(1.7%)
45–64	19734(22.1%)	1221(25.4%)	18513(21.9%)
>=65	67971(76.0%)	3515(73.0%)	64456(79.1%)
Median (IQR)	73.0(65.0–81.0)	71.0(64.0–78.0)	73.0(65.0–81.0)
Report countries (Top 3)			
1	US 110431(72.6%)	US 2119(27.6%)	US 105652(73.1%)
2	CA 9065(6.0%)	JP 1432(18.6%)	CA 8791(6.1%)
3	NL 5298(3.5%)	DE 660(8.6%)	NL 5156(3.6%)
Outcome			
Data available	72937	7593	65344
Death	20300(27.83%)	266(3.5%)	20034(30.6%)
Hospitalized	14925(20.46%)	1013(13.3%)	13912(21.3%)
Life threating	721(0.99%)	37(0.5%)	684(10.5%)
Disability	1693(2.32%)	293(3.9%)	1400(2.1%)
Required intervention	83(0.11%)	16(0.2%)	67(0.1%)
Other outcomes	35215(48.28%)	5968(78.6%)	29247(44.8%)

ONJ, osteonecrosis of the jaw; IQR, interquartile range; US, United States; CA, Canada; NL, Netherland; JP, Japan; DE, Germany.

### 3.2 Disproportionality analysis

**Figure 2:**
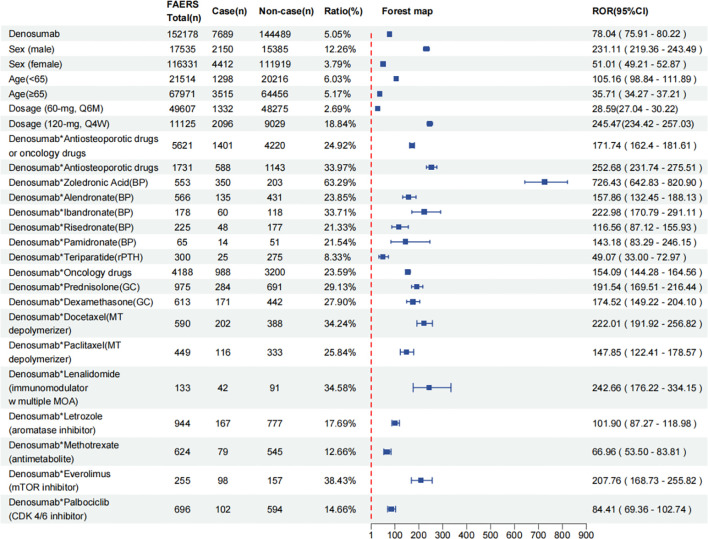
Reported cases and ROR (95% CI) of denosumab-related osteonecrosis of the jaw stratified by age, sex, dosages and integrated medications. ROR, reporting odds ratio; CI, confidence interval; Q4W, every 4 weeks; Q6M, every 6 months; BP, bisphosphonates; rPTH: recombinant parathyroid hormone; GC, glucocorticoids; MT, microtubule; MOA, mechanism of action; mTOR, mechanistic target of rapamycin; CDK, cyclin-dependent kinase.

Disproportionality analysis results from the FAERS database highlight a significant association between denosumab and the risk of ONJ. Among the 152,178 reports related to denosumab, 7,689 cases (5.05%) reported ONJ, yielding a notable ROR of 78.04 (95% CI: 75.91–80.22). As illustrated in **Figure 2**, the RORs and 95% CIs for denosumab-related ONJ were stratified by age, sex, dosage, and combination medications. Male patients exhibited a significantly higher ONJ proportion (12.26% *vs*. 3.79% in females) and a markedly elevated ROR (231.11, 95% CI: 210.45–253.82 *vs*. 51.01, 95% CI: 47.32–54.98). Patients aged<65 years showed a marginally higher risk (ROR = 105.16, 95% CI: 98.84–111.89) compared to those aged ≥65 (ROR = 35.71, 95% CI: 34.27–37.21). The 120 mg Q4w regimen was associated with a substantially higher ONJ proportion (18.8%) compared to the 60 mg Q6m dose (2.69%), as evidenced by RORs of 245.47 (95% CI: 234.42–257.03) and 28.59 (95% CI: 27.04–30.22), respectively. Combination therapies posed the greatest risks, particularly with zoledronic acid (ROR = 726.43, 95% CI: 642.83–820.90), followed by anti-osteoporotic drugs (ROR = 252.68, 95% CI: 231.74–275.51), lenalidomide (ROR = 242.66, 95% CI: 176.22–334.15), ibandronate (ROR = 222.98, 95% CI: 170.79–291.11), docetaxel (ROR = 222.01, 95% CI: 191.92–256.82), and everolimus (ROR = 207.76, 95% CI: 168.73–255.82). Additionally, the concomitant use of denosumab with other drugs was associated with varying increases in ONJ reporting rates. Detailed data on specific drug combinations and their association with ONJ risk are presented in **Figure 2**.

### 3.3 Logistic regression analysis

After excluding reports with missing demographic or dosage data, 44,379 denosumab-related cases (comprising 2,920 ONJ cases and 41,459 non-ONJ AEs) from the initial 152,178 reports were analyzed using logistic regression. This case-control framework facilitates direct comparison between patients who developed ONJ and those experiencing other AEs during denosumab treatment. Univariate analysis identified male sex (*P<*0.001), high-dose administration (*P<*0.001), and concomitant medication use (*P<*0.001) as significant risk factors for denosumab-related ONJ. Although age showed no univariate significance (*P* = 0.120), it was included in multivariable modelling to address potential baseline confounding (**Table 2**).

**Table 2:** Multivariable logistic regression model for risk factors of denosumab-related osteonecrosis of the jaw.

Variables	Crude OR (95%CI)	*P*-value	Adjusted OR (95%CI)^Δ^	*P*-value
Sex (male)	3.08(2.84-3.34)	<0.001		
Age (≥65)	0.94(0.86-1.02)	0.120	1.48(1.34-1.62)	<0.001
Dosage (120 mg, Q4w)	8.03(7.41-8.69)	<0.001	7.18(6.49-7.94)	<0.001
Denosumab*Zoledronic Acid	37.00(28.93-47.32)	<0.001	10.80(8.27-14.10)	<0.001
Denosumab*Alendronate	7.24(5.56-9.43)	<0.001	9.43(6.95-12.80)	<0.001
Denosumab*Ibandronate	9.40(6.16-14.33)	<0.001	5.99(3.51-10.25)	<0.001
Denosumab*Risedronate	4.19(2.77-6.35)	<0.001	3.17(1.87-5.39)	<0.001
Denosumab*Pamidronate	4.38(1.98-9.68)	<0.001		
Denosumab*Teriparatide	2.44(1.48-4.03)	<0.001	2.39(1.31-4.35)	0.005
Denosumab*Prednisolone	7.07(5.97-8.36)	<0.001	3.93(3.20-4.83)	<0.001
Denosumab*Dexamethasone	7.34(5.95-9.05)	<0.001		
Denosumab*Docetaxel	12.60(10.09-15.73)	<0.001	2.81(2.19-3.61)	<0.001
Denosumab*Paclitaxel	8.83(6.74-11.57)	<0.001	2.07(1.51-2.84)	<0.001
Denosumab*Lenalidomide	11.54(6.81-19.57)	<0.001	3.02(1.65-5.55)	<0.001
Denosumab*Letrozole	5.43(4.32-6.81)	<0.001	1.77(1.33-2.34)	<0.001
Denosumab*Methotrexate	3.31(2.49-4.40)	<0.001	3.32(2.37-4.67)	<0.001
Denosumab*Everolimus	14.48(10.13-20.69)	<0.001	4.06(2.72-6.04)	<0.001
Denosumab*Palbociclib	3.11(2.01-4.79)	<0.001		

^Δ^The model simultaneously adjusted for all variables presented in this table; OR, odds ratio; CI, confidence interval; Q4w, every 4 weeks.

The asterisk symbol (*) denotes cases where denosumab was used in combination with or sequentially following another relevant medication.

After adjusting for confounding factors, multivariate logistic regression analysis revealed that age ≥65 years (adjusted odds ratio [aOR] = 1.48, 95% CI: 1.34–1.62, *P<* 0.001), high-dose denosumab (120 mg) (aOR = 7.18, 95% CI: 6.49–7.94, *P<* 0.001), and combination medications were independent risk factors for denosumab-related ONJ. Compared to denosumab monotherapy, the concomitant use of denosumab with other medications significantly increased the risk of ONJ. The most pronounced association emerged with zoledronic acid coadministration (aOR=10.80, 95% CI:8.27–14.10, *P<*0.001). Other combinations that significantly elevated ONJ risk included denosumab with alendronate (aOR = 9.43, 95% CI: 6.95–12.80), ibandronate (aOR = 5.99, 95% CI: 3.51–10.25), risedronate (aOR = 3.17, 95% CI: 1.87–5.39), teriparatide (aOR = 2.39, 95% CI: 1.31–4.35), prednisolone (aOR = 3.93, 95% CI: 3.20–4.83), docetaxel (aOR = 2.81, 95% CI: 2.19–3.61), paclitaxel (aOR = 2.07, 95% CI: 1.51–2.84), lenalidomide (aOR = 3.02, 95% CI: 1.65–5.55), letrozole (aOR = 1.77, 95% CI: 1.33–2.34), methotrexate (aOR = 3.32, 95% CI: 2.37–4.67), and everolimus (aOR = 4.06, 95% CI: 2.72–6.04). All associations reached statistical significance (*P* ≤0.005) as detailed in **Table 2**.

### 3.4 Time‐to‐onset analysis

**Figure 3:**
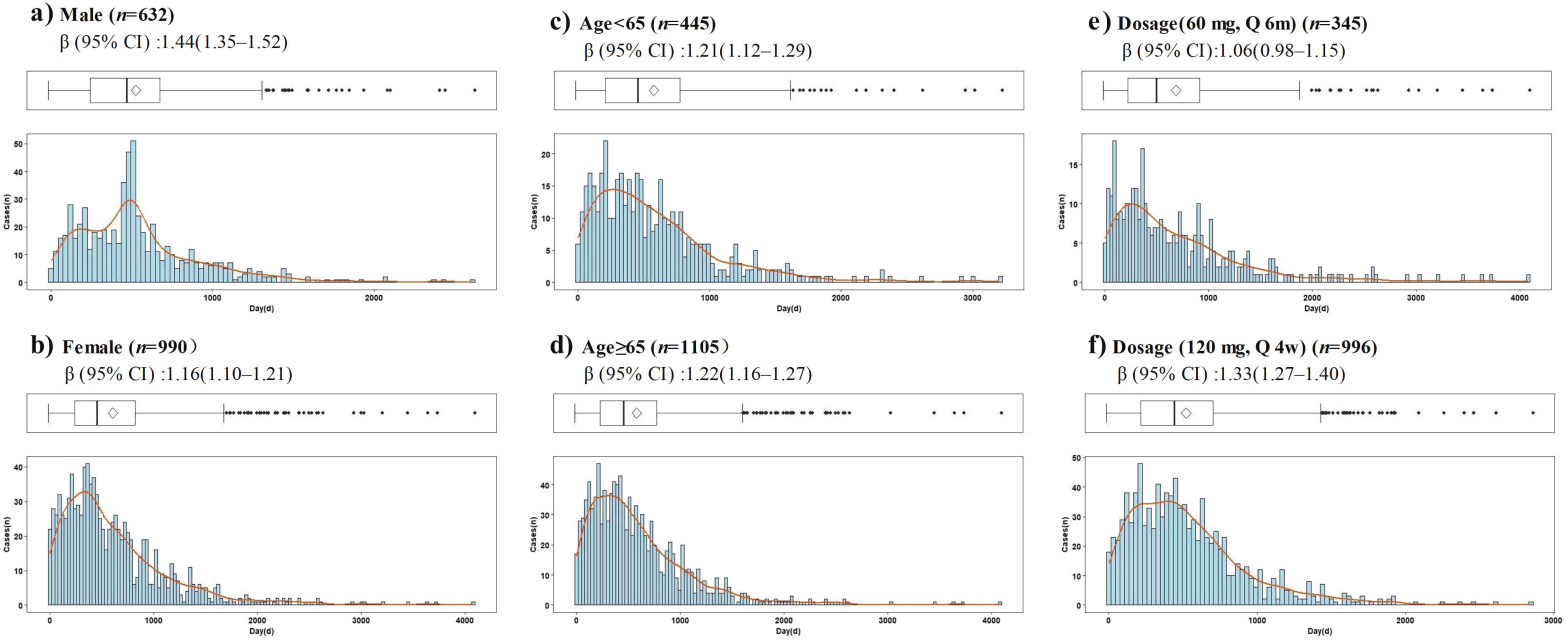
Histogram and Weibull shape parameter of denosumab-related osteonecrosis of the jaw stratified by age, sex, dosages. Q4w, every 4 weeks; Q6m, every 6 months. **(a)** Male, **(b)** Female, **(c)** Age<65, **(d)** Age≥65, **(e)** Dosage(60mg, Q6m), **(f)** Dosge(120mg, Q4w).

**Figure 4:**
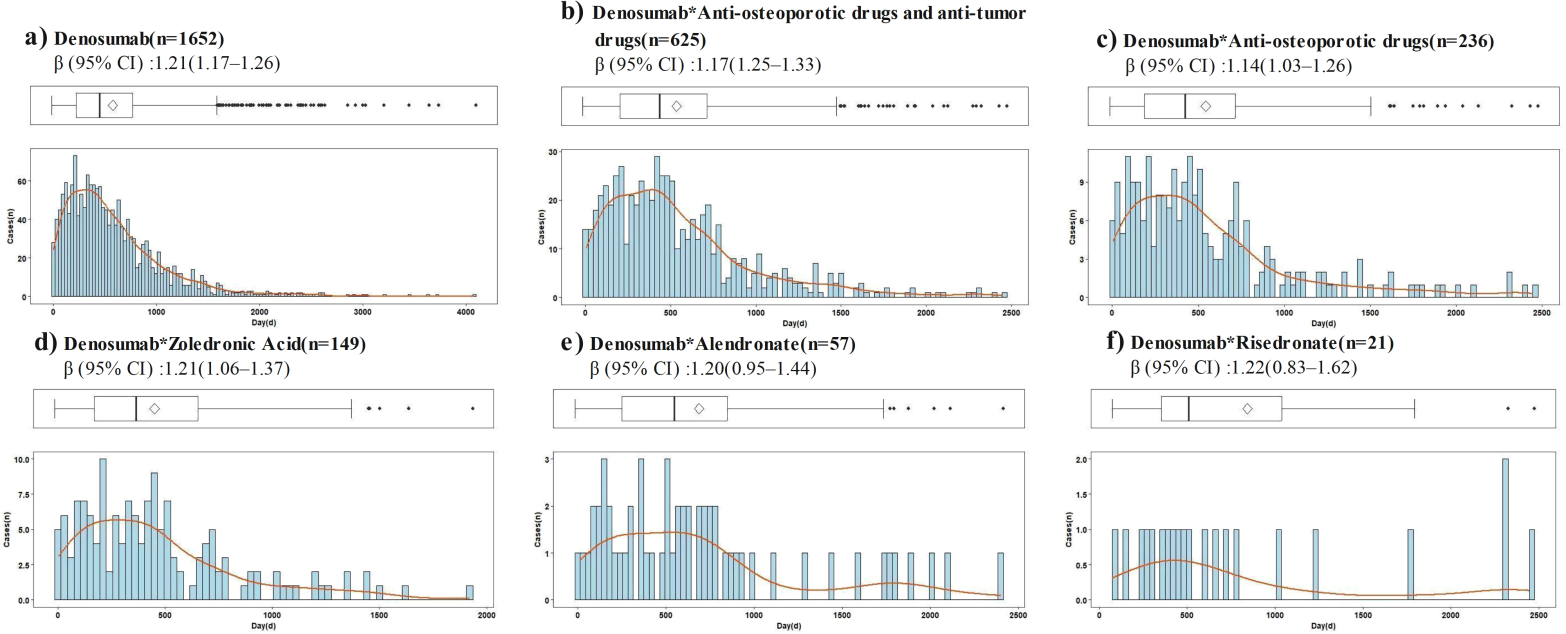
Histogram and Weibull shape parameter of osteonecrosis of the jaw associated with denosumab and its sequential therapy. **(a)** Denosumab, **(b)** Denosumab*Anti-osteoporotic drugs and anti-tumor drugs, **(c)** Denosumab*Anti-osteoporotic drugs, **(d)** Denosumab*Zoledronic Acid, **(e)** Denosumab*Alendronate, **(f)** Denosumab*Risedronate.

**Figure 5:**
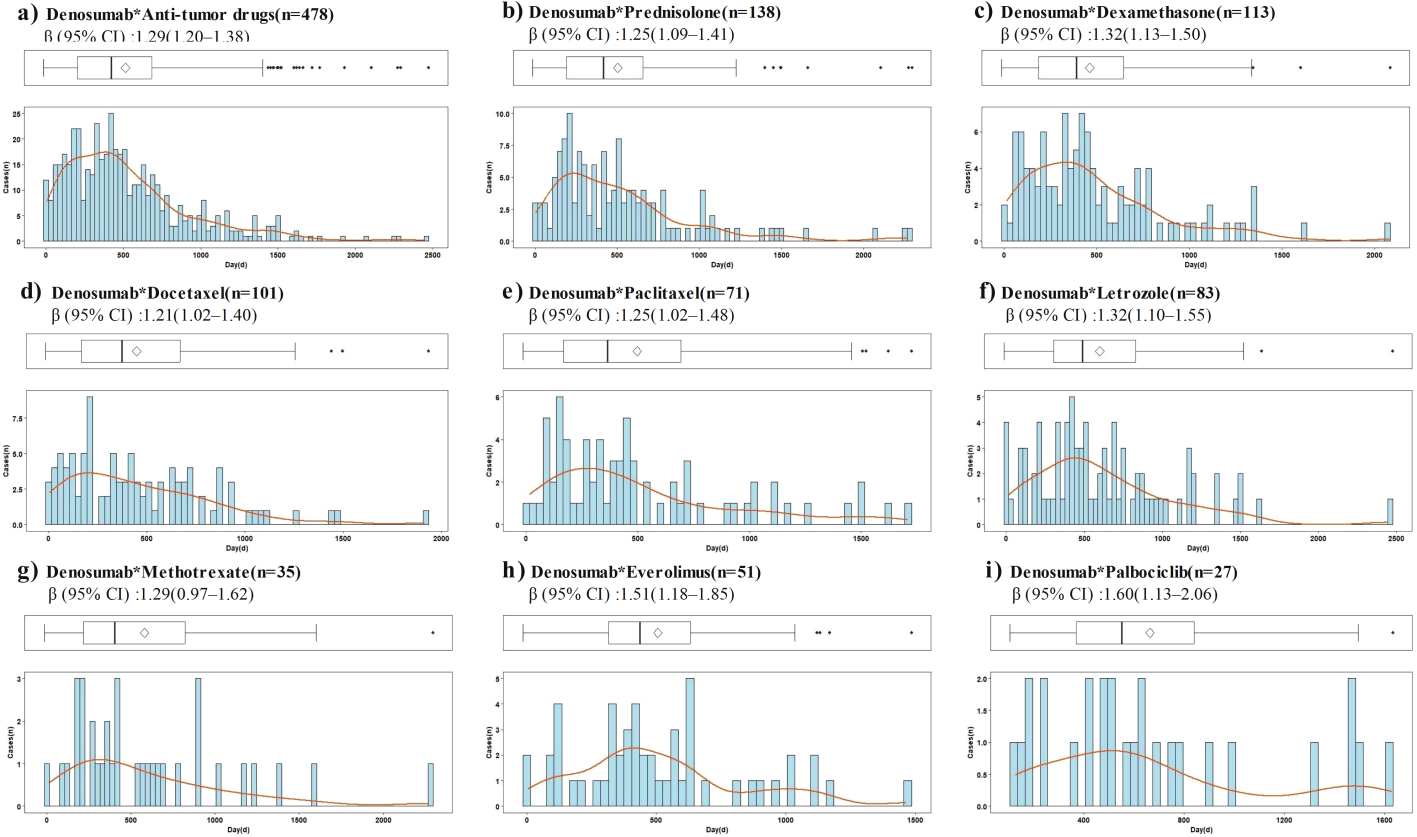
Histogram and Weibull shape parameter of osteonecrosis of the jaw associated with dinomumab and its combination therapy. **(a)** Denosumab*Anti-tumor drugs, **(b)** Denosumab*Prednisolone, **(c)** Denosumab*Dexamethasone, **(d)** Denosumab*Docetaxel, **(e)** Denosumab*Paclitaxel, **(f)** Denosumab*Letrozole, **(g)** Denosumab*Methotrexate, **(h)** Denosumab*Everolimus, **(i)** Denosumab*Palbociclib.

Time-to-onset data were available for 1,652 of 7,689 reports of denosumab-related ONJ. The median time to ONJ onset for denosumab monotherapy was 462.0 days (IQR: 236.0–777.8). Stratified analyses by age, sex, dosage, and concomitant medications consistently demonstrated onset times exceeding 365 days across all subgroups (**Table 3**). In the WSP analysis of denosumab-related ONJ, most subgroups exhibited a wear-out-failure type pattern, with shape parameters (β) and their 95% CIs exceeding 1, indicating an increasing risk of ONJ over time. However, random-failure type patterns were observed in the 60 mg dose group and subgroups involving combinations with alendronate, ibandronate, risedronate, and methotrexate (**Table 3**). Histograms and Weibull parameters stratified by age, sex, dosage, and concomitant medications are illustrated in **Figures 3**–**5**.

**Table 3:** Time-to-onset analysis of denosumab-related osteonecrosis of the jaw stratified by age, sex, dosages and integrated medications.

Stratification variables	Case (n)	Median (IQR), days	*P*-value	Scale parameter: α(95% CI)	Shape parameter: β(95% CI)	Patterns
Denosumab	1652	462.0(236.0–777.8)	–	624.94(598.80–651.09)	1.21(1.17–1.26)	Wear‐out failure
Sex (male)	632	481.0(257.0–682.0)	0.251	590.18(556.48–623.88)	1.44(1.35–1.52)	Wear‐out failure
Sex (female)	990	462.5(247.8–829.3)		650.37(613.58–687.16)	1.16(1.10–1.21)	Wear‐out failure
Age (<65)	445	464.0(221.0–782.5)	0.808	622.23(571.84–672.62)	1.21(1.12–1.29)	Wear‐out failure
Age (≥65)	1105	463.0(238.0–787.0)		629.38(597.29–661.48)	1.22(1.16–1.27)	Wear‐out failure
Dosage (60 mg, Q6m)	345	506.0(234.5–924.5)	0.011a	712.83(638.38–787.29)	1.06(0.98–1.15)	Random failure
Dosage (120 mg, Q4w)	996	452.0(230.0–712.5)		576.47(548.22–604.72)	1.33(1.27–1.40)	Wear‐out failure
Denosumab*Antiosteoporosis and oncological drugs	625	447.0(219.0–719.5)	0.025a	544.17(582.61–621.04)	1.17(1.25–1.33)	Wear‐out failure
Denosumab*Antiosteoporotic drugs	236	435.0(201.5–721.0)	0.089	578.43(510.45–646.40)	1.14(1.03–1.26)	Wear‐out failure
Denosumab*Zoledronic Acid	149	378.0(183.0–665.5)	0.001a	491.80(423.42–560.18)	1.21(1.06–1.37)	Wear‐out failure
Denosumab*Alendronate	57	569.0(263.0–879.5)	0.164	750.39(578.91–921.86)	1.20(0.95–1.44)	Random failure
Denosumab*Ibandronate	19	516.0(307.0–1075.0)	0.390	767.43(409.18–1125.69)	1.01(0.64–1.38)	Random failure
Denosumab*Risedronate	21	504.0(321.5–1128.0)	0.161	895.51(563.21–1227.82)	1.22(0.83–1.62)	Random failure
Denosumab*Oncological drugs	478	434.0(217.3–693.5)	0.014a	565.13(523.83–606.44)	1.29(1.20–1.38)	Wear‐out failure
Denosumab*Prednisolone	138	427.5(204.8–667.8)	0.087	553.00(475.48–630.53)	1.25(1.09–1.41)	Wear‐out failure
Denosumab*Dexamethasone	113	413.0(207.0–673.5)	0.040a	525.76(448.12–603.39)	1.32(1.13–1.50)	Wear‐out failure
Denosumab*Docetaxel	101	384.0(182.0–678.5)	0.010a	486.38(404.22–568.54)	1.21(1.02–1.40)	Wear‐out failure
Denosumab*Paclitaxel	71	383.0(189.0–724.0)	0.164	550.38(442.27–658.50)	1.25(1.02–1.48)	Wear‐out failure
Denosumab*Lenalidomide	19	488.0(379.0–715.0)	0.663	634.26(447.16–821.36)	1.60(1.06–2.15)	Wear‐out failure
Denosumab*Letrozole	83	506.0(319.0–839.0)	0.257	661.16(548.52–773.80)	1.32(1.10–1.55)	Wear‐out failure
Denosumab*Methotrexate	35	422.0(224.0–888.0)	0.896	645.71(471.10–820.32)	1.29(0.97–1.62)	Random failure
Denosumab*Everolimus	51	444.0(321.0–637.0)	0.744	561.29(455.21–667.37)	1.51(1.18–1.85)	Wear‐out failure
Denosumab*Palbociclib	27	563.0(359.0–913.0)	0.161	755.21(566.57–943.85)	1.60(1.13–2.06)	Wear‐out failure

IQR, interquartile range; Q4w, every 4 weeks; Q6m, every 6 months. ^a^. *P*<0.05.

The asterisk symbol (*) denotes cases where denosumab was used in combination with or sequentially following another relevant medication.

**Figure 6:**
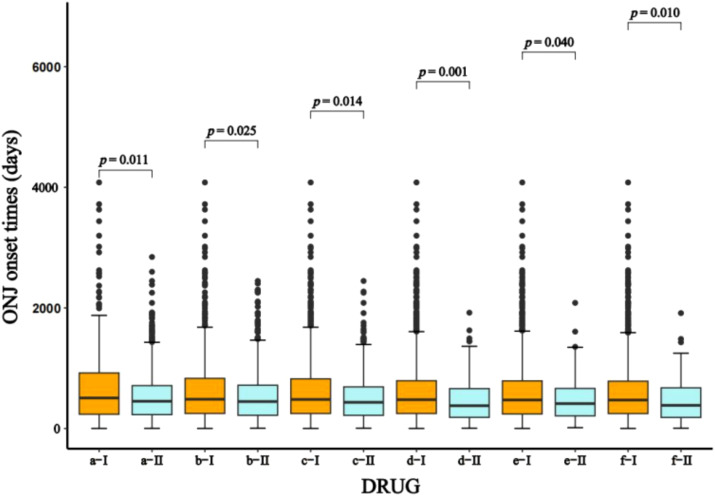
The time-to-onset of ONJ caused by different doses and combinations of different drugs with denosumab. ONJ: osteonecrosis of the jaw; a-I: Denosumab(60mg, Q6m); a-II: Denosumab(120mg, Q4w); b-I: Denosumab without Antiosteoporotic drugs and anticancer drugs; b-II: Denosumab*Anti-osteoporotic drugs and anti-tumor drugs; c-I: Denosumab without Anti-tumor drugs; c-II: Denosumab*Anti-tumor drugs; d-I: Denosumab without Zoledronic Acid; d-II: Denosumab*Zoledronic Acid; e-I: Denosumab without Dexamethasone; e-II: Denosumab*Dexamethasone; f-I: Denosumab without Docetaxel; f-II: Denosumab*Docetaxel.

No significant differences in the time to ONJ onset were observed between sex (*P* = 0.251) or age groups (*P* = 0.808). However, significant differences were noted for dosage and combination medications (**Table 3**). The median time to ONJ onset was significantly longer in the 60 mg dose group (506.0 days, IQR: 234.5–924.5) compared to the 120 mg dose group (452.0 days, IQR: 230.0–712.5) (*P* = 0.011), suggesting that higher doses may accelerate ONJ onset. The median time to ONJ onset was 447.0 days (IQR: 219.0–719.5) when denosumab was combined with anti-osteoporotic or anti-tumor drugs, which was significantly shorter compared to denosumab without anti-osteoporotic drugs and anticancer drugs (*P* = 0.025). Specific combinations further reduced the time to onset: co-administration with zoledronic acid resulted in a median onset of 378.0 days (IQR: 183.0–665.5, *P* = 0.001), anti-tumor drugs 434.0 days (IQR: 217.3–693.5, *P* = 0.014), dexamethasone 413.0 days (IQR: 207.0–673.5, *P* = 0.040), and docetaxel 384.0 days (IQR: 182.0–678.5, *P* = 0.010) (**Table 3**). Box plots (**Figure 6**) further validated these findings, highlighting the significant impact of dosage and combination therapies on the time to ONJ onset.

## 4 Discussion

This comprehensive pharmacovigilance study, leveraging data from the FAERS database, provides critical insights into the risk factors and onset time of denosumab-related ONJ. The findings underscore the multifactorial nature of ONJ pathogenesis, with significant contributions from dosage, concomitant medications, and patient demographics. By integrating disproportionality analysis, logistic regression, and time-to-onset assessments, this study advances our understanding of the interplay between denosumab and its clinical environment, offering actionable strategies for risk mitigation.

The disproportionality analysis demonstrated a robust association between denosumab and ONJ, with a ROR of 78.04 (95% CI: 75.91–80.22), consistent with previous studies (12, 18). This heightened propensity for ONJ is likely attributed to denosumab’s potent inhibition of osteoclast activity through RANKL suppression (29). However, our analysis extends these observations by quantifying the dose-dependent risk profile. The 120 mg Q4w regimen, which is mainly utilized in oncology settings, exhibited a significantly higher ROR of 245.47 in comparison to 28.59 for the 60 mg Q6m regimen. Moreover, it had a higher incidence proportion (18.41% *vs*. 2.69%), which corroborates the clinical reports indicating that cumulative exposure and higher dosing levels intensify the inhibition of osteoclasts, thus making it more likely to lead to ONJ (19). This dose-effect relationship was further validated by time-to-onset analyses, where the 120 mg Q4w regimen accelerated median ONJ onset by 54 days compared to the 60 mg Q6m regimen (*P* = 0.011). This finding is clinically significant, as higher doses are commonly prescribed for malignancy-related bone complications. Clinicians must weigh the benefits of aggressive dosing against the heightened ONJ risk, particularly in patients with pre-existing dental vulnerabilities.

The most striking risk amplification emerged from combination therapies, particularly with zoledronic acid, which yielded an ROR of 726.43 (95% CI: 642.83–820.90)—a magnitude exceeding prior estimate (12). These findings corroborate the observations by Hasegawa et al. (18), whose analysis of the Japanese Adverse Drug Event Report database identified a heightened risk of early-onset ONJ in patients transitioning from zoledronic acid to denosumab therapy. In our cohort, the median time to ONJ onset was approximately three months shorter for denosumab-zoledronic acid combinations (378.0 days, IQR: 183.0–665.5) compared to denosumab monotherapy (462.0 days, IQR: 236.0–777.8), further underscoring the accelerated hazard profile. Mechanistically, this synergistic risk likely arises from dual osteoclast suppression: denosumab inhibits RANKL-mediated osteoclastogenesis, while zoledronic acid disrupts the mevalonate pathway, collectively compounding skeletal toxicity and impairing bone remodeling (29, 30). Additionally, zoledronic acid’s long half-life and high affinity for bone hydroxyapatite result in prolonged skeletal accumulation (31). When transitioning from zoledronic acid to denosumab, the residual effects of zoledronic acid may lead to overlapping pharmacological actions, resulting in enhanced osteoclast suppression and an elevated risk of medication-related ONJ (MRONJ). Similarly, combinations with glucocorticoids (e.g., prednisolone: ROR = 191.54) or cytotoxic agents (e.g., docetaxel: ROR = 222.01) suggest overlapping mechanisms, such as impaired angiogenesis and delayed mucosal healing (32, 33). These results align with multivariate logistic regression findings, where aORs for combinations like Denosumab*Zoledronic acid (aOR = 10.80), Denosumab*Prednisolone (aOR = 3.93) and Denosumab*Docetaxel (aOR = 2.81) remained robust after confounder adjustment. Collectively, these findings underscore the critical imperative to avoid overlapping antiresorptive therapies and prioritize non-bisphosphonate alternatives in high-risk patients, as combination medications—particularly with bisphosphonates, glucocorticoids, or cytotoxic agents—significantly hasten the onset of denosumab-related ONJ, emphasizing the urgent need for vigilant monitoring in these vulnerable cohorts.

While the effects of age and sex were less pronounced compared to pharmacological factors, their roles remain noteworthy. Male patients demonstrated a threefold higher risk of ONJ than females (ROR = 231.11 *vs*. 51.01), a disparity consistent with previous studies that associate male sex with delayed diagnosis and higher comorbidity burdens (32). In contrast, age ≥65 years showed only a marginal risk elevation (aOR = 1.48), challenging the hypothesis that age-related declines in bone turnover predominantly drive ONJ pathogenesis (32, 34). This is further supported by time-to-onset analyses, where ageing minimally influenced latency periods (*P* = 0.808), suggesting that pharmacological variables—such as high-dose regimens or polypharmacy—and iatrogenic factors (e.g., invasive dental procedures) dominate risk trajectories. The attenuated demographic effect likely reflects confounding by clinical practice patterns: older patients are more frequently prescribed intensive therapies (e.g., 120 mg denosumab with zoledronic acid), which mask baseline age-related risks. These findings underscore the necessity of rigorous confounder adjustment in ONJ risk stratification, ensuring that demographic associations are not conflated with treatment intensity or drug interactions.

The analysis revealed a prolonged median time-to-onset of 462 days for denosumab-related ONJ, indicating a cumulative risk profile associated with sustained suppression of bone remodeling. This finding, however, does not contradict guideline recommendations for scheduling invasive dental procedures just before the next dose or employing short-term drug holidays (32, 35). These strategies are designed to mitigate the acute risk during the critical wound-healing phase following an invasive procedure by leveraging denosumab’s shorter pharmacokinetic half-life, despite its prolonged pharmacodynamic effect. Our data on long latency complement these guidelines by emphasizing that the overall risk is long-term and cumulative. Therefore, while short-term interventions are valuable for planned procedures, ongoing dental vigilance is essential throughout the treatment course, especially for patients receiving long-term therapy (34). Further temporal analysis using the Weibull shape parameter (β) revealed distinct hazard patterns for ONJ onset. For most subgroups, β > 1 indicated a wear-out-failure pattern, signifying an escalating ONJ risk over time. This aligns with denosumab’s mechanism of cumulative osteoclast suppression, where prolonged exposure exacerbates skeletal vulnerability (29, 32). Exceptions included the 60 mg dose group and combinations such as Denosumab*Methotrexate or Denosumab*Risedronate, which exhibited random-failure patterns (β ≈ 1), suggesting stochastic risk unaffected by treatment duration. These divergent patterns may reflect heterogeneous pharmacodynamic interactions—for instance, methotrexate’s anti-angiogenic effects could override time-dependent osteoclast inhibition, leading to unpredictable risk trajectories (36, 37). The prolonged latency period (median onset: 462 days for monotherapy) further underscores the insidious nature of denosumab-related ONJ, where risk accrues subtly but persistently. Clinically, these findings emphasize the need to tailor monitoring protocols: while most patients require long-term vigilance due to cumulative hazard, subgroups with random-failure patterns demand heightened suspicion irrespective of treatment duration (34). Such stratification could optimize early detection and mitigate the morbidity associated with this debilitating AE.

This study provides crucial evidence for optimizing denosumab therapy, demonstrating that effective risk mitigation requires comprehensive consideration of pharmacological, demographic and clinical factors. Clinicians must conduct comprehensive, individualized risk assessments for each patient considering: (i) indication-specific profiles (e.g., oncology *vs*. osteoporosis indications), (ii) current and planned antiresorptive therapy (including prior bisphosphonate exposure duration), (iii) demographic factors (particularly age ≥65 years), and (iv) baseline oral health status—with special attention to those requiring invasive dental procedures. High-signal populations—particularly elderly oncology patients undergoing sequential zoledronic acid to denosumab therapy—require pre-emptive dental evaluations and biannual oral examinations to detect early signs of ONJ. Through systematic implementation of these evidence-based strategies—incorporating dose optimization, targeted monitoring protocols, and preventive dental care—healthcare teams can maximize denosumab’s clinical benefits while substantially reducing the incidence and severity of medication-related ONJ.

This study has several limitations inherent to the pharmacovigilance design and FAERS database. First, reliance on spontaneous reporting introduces potential underrepresentation of true ONJ incidence due to underreporting or incomplete data, particularly regarding baseline patient characteristics such as comorbidities, treatment duration, cumulative dose, disease severity, dental history, and prior invasive procedures—factors that may confound risk assessment. Although we focused on pre-specified concomitant medications and patient factors based on biological plausibility and clinical guidance, this hypothesis-driven approach did not include a fully agnostic, data-driven exploration (e.g., machine learning or broad regression screening of all FAERS codes) that might have revealed previously unrecognized risk factors. Future studies employing such hypothesis-free methods could build valuably on our findings to generate new insights into denosumab-related ONJ. Second, the absence of granular clinical details (e.g., longitudinal treatment history, sequential drug use, exact protocols) precluded analysis of regimen-specific risk variations, complex dosing patterns like drug holidays, and the polypharmacy interplay in ONJ pathogenesis. Third, confounding by indication cannot be excluded; patients receiving high-risk combinations (e.g., denosumab with zoledronic acid) often present with advanced disease, complicating causal inference. Furthermore, as with all observational studies, residual confounding remains a possibility despite our multivariate adjustments. Unmeasured or unknown factors that were not captured in the FAERS database could influence the observed associations. Finally, the observational design inherently limits the establishment of causality. To address these gaps, future research should adopt multicenter, prospective randomized controlled trials to validate the impact of denosumab—both as monotherapy and in combination—on ONJ risk, while rigorously controlling for confounders. Concurrently, mechanistic studies are needed to elucidate how drug interactions (e.g., denosumab with anti-angiogenic agents) perturb bone metabolism and healing pathways. Additionally, individualized risk stratification models, incorporating genetic, demographic, and treatment variables, should be developed to guide precision prescribing and mitigate morbidity in high-risk populations.

## 5 Conclusions

This study establishes that denosumab-related ONJ risk is significantly influenced by dosage, concomitant medications, and patient demographics. The 120 mg dose and combinations with bisphosphonates or glucocorticoids markedly accelerate ONJ onset, necessitating stringent risk mitigation strategies. Clinicians should prioritize preemptive dental evaluations in high-risk cohorts (e.g., elderly patients on high-dose denosumab or polypharmacy) and exercise caution when combining antiresorptives. Future research should explore mechanistic pathways underlying drug-drug interactions and validate these findings in prospective cohorts to refine risk prediction models.

## Data Availability

The original contributions presented in the study are included in the article/**Supplementary Material**. Further inquiries can be directed to the corresponding author.
